# Universal scaling law: transcription factors homeostatically control promoter activity

**DOI:** 10.1038/s41392-025-02519-x

**Published:** 2026-01-08

**Authors:** Minhao Gao, Aimin Wu, Min Wu

**Affiliations:** 1 Key Laboratory of Orthopaedics of Zhejiang Province, The Second Affiliated Hospital and Yuying Children’s Hospital of Wenzhou Medical University, Wenzhou, Zhejiang China; 2 Joint Research Centre on Medicine, The Affiliated Xiangshan Hospital of Wenzhou Medical University, Ningbo, China and Tianfu Jincheng Laboratory, Chengdu, China; 3 Wenzhou Institute, University of Chinese Academy of Sciences, Wenzhou, China

**Keywords:** RNA splicing, Genomic instability

A recent study published in *Science* by Parisutham et al.^1^ revealed a universal principle governing transcription factor (TF) function in *E. coli*, demonstrating that highly diverse TFs regulate gene expression through a common stabilizing mechanism with RNA polymerase (RNAP). This mechanism uniquely renders an inverse scaling relationship between TF-mediated fold change and constitutive promoter strength, enabling homeostatic buffering of gene expression against genetic and environmental perturbations. These fundamental findings challenge classical models of TF action unexpectedly^2^ and offer a unified framework for predicting transcriptional regulation across biological contexts.

Gene expression is precisely controlled by TFs that activate or repress target promoters through mechanisms often described as context dependent.^3^ A long-standing puzzle in molecular biology is that the same TF can function as an activator for one promoter and a repressor for another, even in similar and perhaps the same genomic contexts. The precise biophysical mechanisms governing this intriguing promoter-dependent functional switch have remained elusive for decades, and studies have often been hampered by the complexity of in vivo systems where multiple variables exist and are altered simultaneously. Parisutham et al. systematically approached this issue and dissected the potentially fundamental rule by constructing a controlled experimental system to masterfully pinpoint the relationship between TF function and promoter identity.

The authors measured fold-changes in gene expression for eight different TFs—including activators (CpxR, MetR, and SoxS) and repressors (LacI, MngR, PdhR, AscG, and AcrR)—across a comprehensive, high-diversity library of synthetic promoters with varying strengths created by mutating the -35 region. Strikingly, they observed a consistent inverse relationship for all TFs: stronger promoters exhibited substantially diminished regulatory effects. This scaling behavior appears to boast a remarkable dynamic range of regulation, from 100-fold activation to 1000-fold repression, and was independent of the regulatory function of TFs or their binding location relative to the promoter.

**Fig. 1:**
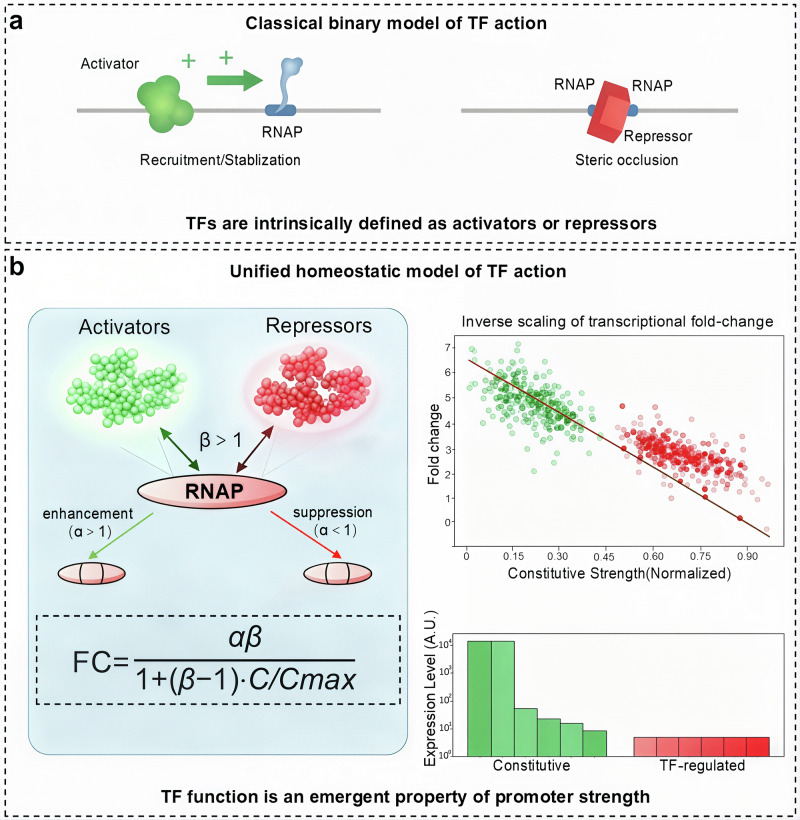
A universal inverse scaling law underlies the critical homeostatic feature in transcriptional regulation. **a** The classical binary model of TF action. Traditionally, transcription factors are intrinsically classified as either activators or repressors. Activators (left) are thought to function primarily by recruiting or stabilizing RNA polymerase (RNAP) at the promoter. In contrast, repressors (right) are often modeled as functioning through steric hindrance, physically occluding RNAP from binding to the promoter. **b** A unified model in which both activators and repressors in *E. coli* function primarily by stabilizing RNA polymerase (RNAP) at the promoter (a mechanism that is parameterized by *β* > 1) is proposed. The net regulatory outcome (activation or repression) is determined by the combined effect on RNAP recruitment (*β*) and transcription initiation (*α*). This preliminarily determined stabilizing mechanism predicts an inverse relationship between the fold change in gene expression and the constitutive strength of the promoter. Stronger promoters exhibit diminished regulatory effects from transcription factors (TFs). Consequently, TFs buffer gene expression against perturbations. While genetic mutations or physiological changes can cause wide variations in constitutive expression, TF-regulated expression levels remain robust and confined to a narrow range, demonstrating homeostatic control

To further explore this universal behavior, the authors employed a thermodynamic model that decouples TF regulation into two steps: RNAP recruitment (parameterized by β) and transcription initiation (parameterized by α).^4^ Fitting their data to this model revealed that all the TFs exerted their functions primarily through stabilizing interactions with RNAP (*β* > 1). This finding directly contradicts the long-held steric hindrance model for repressors, where TF binding physically occludes RNAP access (which would require *β* < 1). The net regulatory outcome, activation or repression, was determined less by the intrinsic identity of the TF and more by the interplay between its universal stabilizing effect (β) and its specific effect on the initiation step (α). A TF with *β* > 1 and *α* > 1 acts as an activator, whereas one with *β* > 1 and *α* < 1 functions as a repressor, elegantly reconciling the apparent functional dichotomy (Fig. 1).

The robustness of this inverse scaling relationship was rigorously tested across diverse perturbation modes. The relationship remained unchanged regardless of whether constitutive expression was altered through genetic mutations in promoter sequences, physiological changes in growth media that affect the global cellular state, or direct modulation of RNAP availability via an orthogonal sigma factor (σ²⁸) system. This finding demonstrates that TFs fundamentally act as strong homeostatic devices that buffer expression levels against perturbations; for example, a 200-fold variation in constitutive expression across promoter mutants was compressed into only a 2-fold variation in TF-regulated expression. Furthermore, this unprecedentedly unifying relationship extended to complex endogenous regulatory architectures, including natural promoters with multiple TF binding sites and DNA looping configurations. By reanalyzing existing data from AraC, CRP, and in vitro CarD studies, the authors showed that all datasets collapse onto a single theoretical curve under appropriate rescaling, reinforcing the breathtaking generality of this stabilizing mechanism across nearly six orders of magnitude in fold-change.

Taken together, the results of this work greatly reframe our understanding of TF function, suggesting that the classic binary classification of TFs as “activators” or “repressors” is secondary to the intrinsic strength of the promoter within a unified stabilizing framework.^5^ The proposed model provides a powerful, predictive tool for the rational design of synthetic genetic circuits and offers fresh insights into evolutionary constraints on regulatory networks. Despite this remarkable discovery, key challenges include elucidating the precise biophysical basis of this ubiquitous stabilization and determining how cofactors and posttranslational modifications tune the parameters *α* and *β*. A paramount question is whether this universal scaling law governs transcriptional regulation in eukaryotes, where chromatin dynamics boasts many more layers of complexity.
